# Sun-induced fluorescence and gross primary productivity during a heat wave

**DOI:** 10.1038/s41598-018-32602-z

**Published:** 2018-09-21

**Authors:** G. Wohlfahrt, K. Gerdel, M. Migliavacca, E. Rotenberg, F. Tatarinov, J. Müller, A. Hammerle, T. Julitta, F. M. Spielmann, D. Yakir

**Affiliations:** 1 University of Innsbruck, 6020 Innsbruck, Austria; 2 Max Planck Institute for Biogeochemistry, 07745 Jena, Germany; 3 Weizmann Institute of Science, 76100 Rehovot, Israel; 4 University of Milano-Bicocca, 20126 Milan, Italy

## Abstract

Remote sensing of sun-induced chlorophyll fluorescence (SIF) has been suggested as a promising approach for probing changes in global terrestrial gross primary productivity (GPP). To date, however, most studies were conducted in situations when/where changes in both SIF and GPP were driven by large changes in the absorbed photosynthetically active radiation (APAR) and phenology. Here we quantified SIF and GPP during a short-term intense heat wave at a Mediterranean pine forest, during which changes in APAR were negligible. GPP decreased linearly during the course of the heat wave, while SIF declined slightly initially and then dropped dramatically during the peak of the heat wave, temporally coinciding with a biochemical impairment of photosynthesis inferred from the increase in the uptake ratio of carbonyl sulfide to carbon dioxide. SIF thus accounted for less than 35% of the variability in GPP and, even though it responded to the impairment of photosynthesis, appears to offer limited potential for quantitatively monitoring GPP during heat waves in the absence of large changes in APAR.

## Introduction

Over the past decade, land ecosystems have removed around one quarter of the carbon emitted by human activities annually, another quarter being removed by the oceans^1^. Without these sinks, global warming would have proceeded at approximately double speed^2^. Whether land ecosystems will continue to significantly remove CO_2_ from the atmosphere or whether human emissions will eventually outpace sinks, is highly uncertain^3,4^, as different carbon cycling models produce widely differing future source/sink estimates^5^. This uncertainty has important practical consequences as the warming relative to pre-industrial times is approximately linearly dependent on cumulative CO_2_ emissions, leaving a finite amount of allowable CO_2_ emissions in order to constrain warming below some threshold^6,7^. The magnitude of the required reduction measures critically depends on the strength of the terrestrial and oceanic sinks, which are projected to decline with realised reductions in atmospheric CO_2_ concentrations^8^. To this end, a robust monitoring system is required which allows quantifying the CO_2_ uptake by land ecosystems at global scale with high spatial and temporal resolution and in response to climate variability and extremes^9^ and helps reduce uncertainties in Earth system models^10^.

In terms of trade-offs between global coverage and spatio-temporal resolution, remote sensing approaches offer the greatest potential for monitoring the CO_2_ uptake by land ecosystems^11^. In particular the use of remotely sensed sun-induced chlorophyll fluorescence (SIF) has recently emerged as a promising approach for tracking vegetation photosynthesis^12^. Solar radiation absorbed by plant chlorophyll molecules has three possible fates: It is either used for generating energy required for photosynthesis (photochemical quenching), dissipated as heat (non-photochemical quenching) or re-emitted as fluorescence at a higher wavelength compared to the absorption^13^. Thus, even though fluorescence competes for the same excitation energy as photosynthesis, their relationship is neither unique nor simple^14^. Nevertheless, SIF has been repeatedly demonstrated to scale with GPP across broad gradients in productivity^15,16^ and/or along the seasonal cycle^17,18^, driven mainly by underlying large changes in the magnitude of absorbed photosynthetically active radiation (APAR)^19^. It remains to be demonstrated, however, how well ecosystem-scale SIF is able to track changes in GPP in situations when APAR is constant, but GPP declines in response to stress. SIF may then decrease in concert with GPP as excess energy is increasingly dissipated via non-photochemical quenching (NPQ), or may also increase if NPQ mechanisms become ineffective and excess energy is emitted as SIF^14,19^. Improving our understanding of the SIF-GPP relationship under stress is particularly relevant, as climate extremes are likely to become more frequent in a future climate^20^.

The objective of this study was thus to investigate the *in-situ* relationship between ecosystem-scale SIF and GPP during a naturally occurring short-term intense heat wave. We hypothesized that, in the absence of changes in APAR, GPP would decrease as a result of diffusional and biochemical limitations in photosynthesis in response to the heat wave and that the excess absorbed energy would be largely dissipated as heat, resulting in little to no change in SIF. To this end we conducted joint ecosystem-scale flux measurements of CO_2_ and carbonyl sulfide, COS - a novel independent proxy for GPP^21,22^, together with measurements of SIF from a flux tower above a semi-arid pine forest in Israel^23^, which is characterized by frequently occurring short-term intense heat waves^24^, and analysed the resulting empirical data in combination with a process-based coupled radiative transfer and photosynthesis model^25^ (see Methods).

## Results

**Figure 1:**
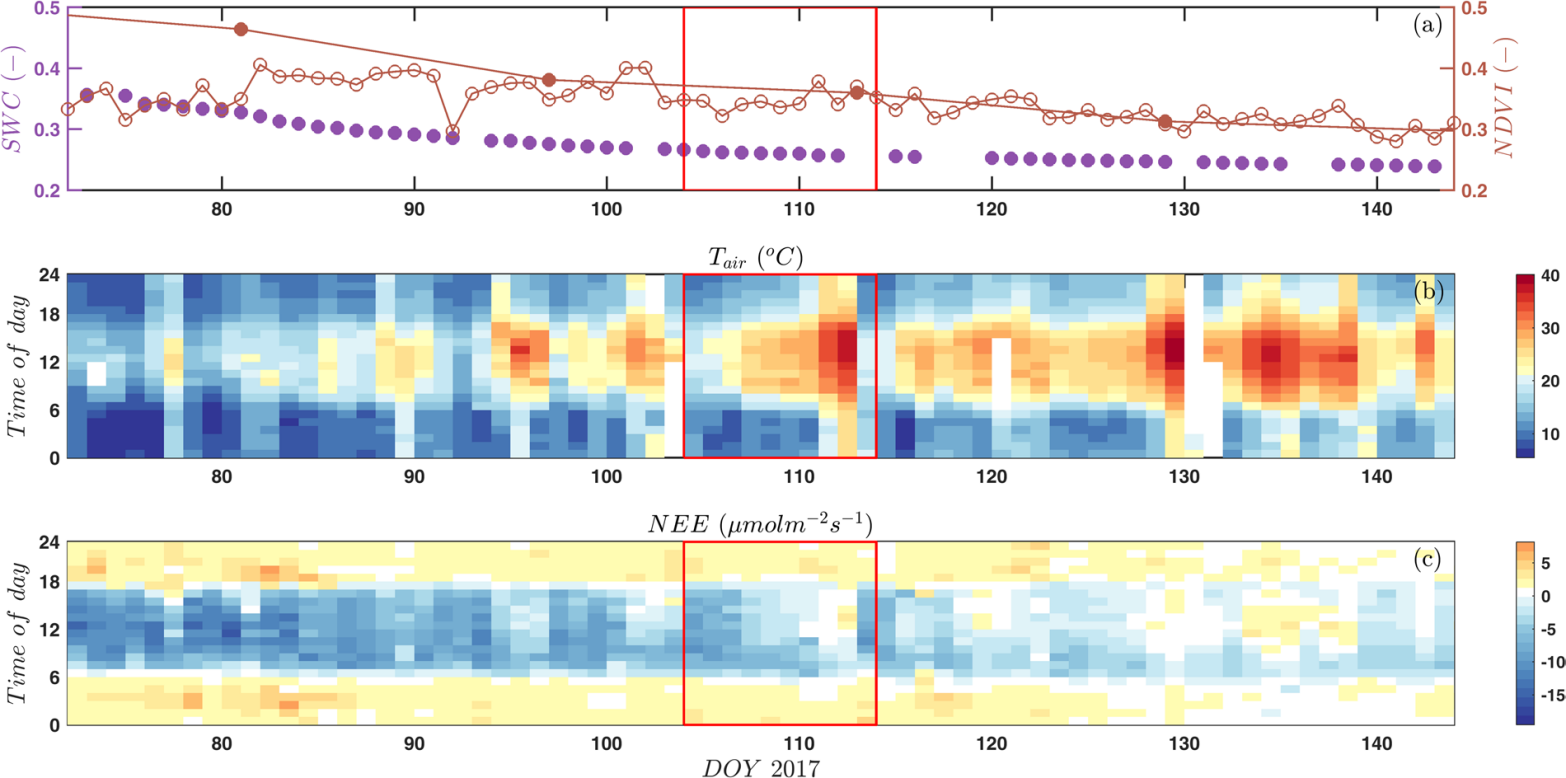
Overview of the environmental conditions and the ecosystem-atmosphere exchange processes during the entire measurement campaign: (**a**) soil water content (SWC, %) and MODIS (closed symbols) and narrow-band tower-based (open symbols) normalized difference vegetation index (NDVI), (**b**) air temperature (T_air_, °C) and (**c**) the gap-filled net ecosystem CO_2_ exchange (NEE, µmol m^−2^ s^−1^). Red rectangles in panels a-c indicate the heat wave period and the first day thereafter, shown in detail in Fig. 2.

During the measurement campaign, from the beginning of March to the end of May 2017, there was a steady decline in soil water content (4.8 mm total precipitation during this period) and the satellite-based (MODIS) normalised difference vegetation index (NDVI), a measure of vegetation greenness (Fig. 1a). Initially, the tower-based NDVI was lower than the satellite-based one (i.e. a stronger contribution of greener vegetation in the larger satellite footprint compared to the radiometric footprint of the narrow-band sensor), but from the beginning of April (DOY 95) on both NDVI time series nicely converged (Fig. 1a), indicating that the tower-based measurements were consistent with the changes observed at the larger scale. Air temperatures increased throughout the measurement campaign, but with a much larger day to day variability (Fig. 1b), characteristic of the high probability of short-term heat waves (termed ‘hamsim’) during spring^24^. During the measurement period, the net CO_2_ uptake decreased and the sink activity progressively shifted towards the early morning hours (Fig. 1c), indicative of the transition towards the summer drought^26^.

**Figure 2:**
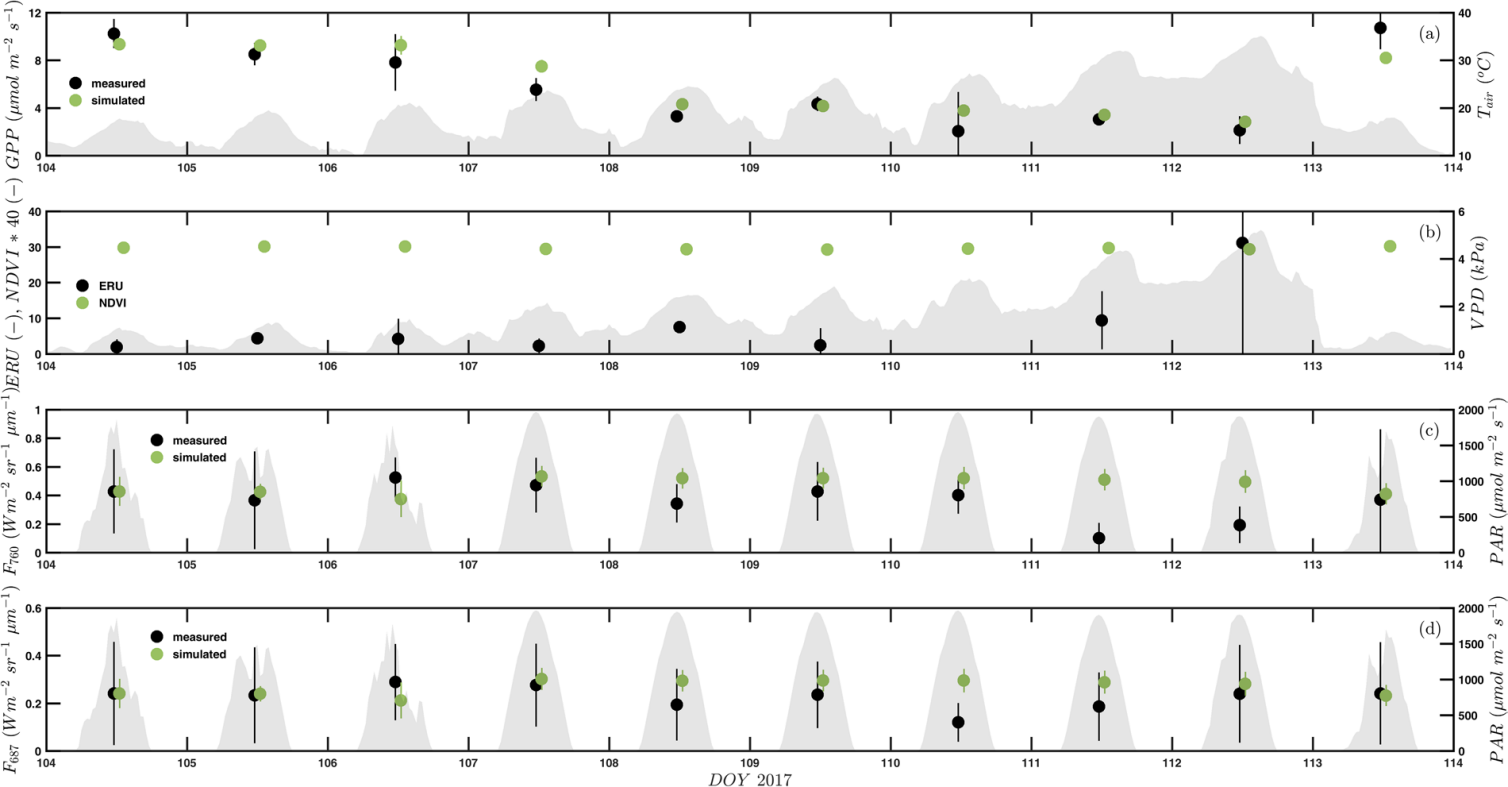
Midday (10–14 local time) mean (±standard deviation) (**a**) inferred gross primary productivity (GPP, µmol m^−2^ s^−1^), (**b**) the ecosystem relative uptake rate (ERU) and the normalised difference vegetation index (NDVI from hyperspectral sensor), and sun-induced fluorescence in the (**c**) O_2_-A (F_760_) and (**d**) O_2_-B (F_687_) band (W m^−2^ sr^−1^ µm^−1^) during the heat wave and the first day thereafter. Hourly air temperature (T_air_, °C), vapour pressure deficit (VPD, kPa) and incident photosynthetically active radiation (PAR, µmol m^−2^ s^−1^) are shown in panels a-d as grey shading. Simulated SIF is scaled to the measured value during the first day of the heat wave (see Methods). Simulated GPP and SIF (green symbols) are slightly offset horizontally from measured values (black symbols) for improved clarity.

On DOY 104 (April 14 2017) a heat wave, which lasted until DOY 112 (April 22 2017), commenced and led to an increase in the daily maximum air temperature from 18 °C (maximum radiometric surface temperature of 20 °C) to 35 °C (maximum radiometric surface temperature of 40 °C) (Fig. 2a). Minimum air temperatures were ca. 10 °C during the early phase of the heat wave and did not fall below 25 °C during the last two days (DOY 111–112; Fig. 2a). Along with air temperature, relative humidity decreased from 43% to 9%, increasing the vapour pressure deficit up to 5 kPa (Fig. 2b). The day following the breakdown of the heat wave (DOY 113), air temperatures and the vapour pressure deficit returned back to values encountered at the start of the heat wave (Fig. 2a,b). Changes in soil water content (Fig. 1a) and vegetation greenness (NDVI from hyperspectral sensor; Fig. 2b) during the course of the heat wave were minimal (1.4% reduction from first to last day of heat wave).

Midday (10–14 local time) mean GPP inferred from flux partitioning^24^ decreased by ca. 80% from ca. 10 µmol m^−2^ s^−1^ to ca. 2 µmol m^−2^ s^−1^ during and returned back to ca. 10 µmol m^−2^ s^−1^ after the end of the heat wave, a trend which is consistent with earlier studies^24^ and was reasonably well captured by the SCOPE model (Fig. 2a), thanks largely to the pre-scribed decrease of V_Cmax_ during the heat wave (see Methods). The ecosystem-scale CO_2_ (*F*_*CO2*_) and COS (*F*_*COS*_) flux measurements, together with the corresponding mixing ratios (*X*_*CO2*_ and *X*_*COS*_) were used to calculate the so-called ecosystem relative uptake rate (ERU)^22^:

1 $$ERU=\frac{{F}_{COS}}{{X}_{COS}}/\frac{{F}_{CO2}}{{X}_{CO2}}.$$

The ERU remained relatively constant (3.9 ± 2.1), with the exception of the last day of the heat wave (DOY 112), when it increased sharply (Fig. 2b; no COS flux data gathered on the day following the end of the heat wave), indicating a shift from diffusional to biochemical limitation of photosynthesis.

Midday mean F_760_ was roughly constant during the first seven days of the heatwave (DOY 104–110), followed by a dramatic decrease (on average by 70%) during the last two days (DOY 111–112) and recovery to pre-heat wave values on the day after the end of the heat wave (DOY 113; Fig. 2c). Measured F_760_ was much more variable during midday of the first three days of the heat wave (DOY 104–106) and at the day after the end of the heat wave (DOY 113), due to variable insulation (Fig. 2c). Midday mean F_687_ was relatively constant during the entire heat wave, except for a strong decrease (by 50%) on the seventh day (DOY 110) (Fig. 2d). The variability of measured F_687_ was comparatively high even during perfect clear sky days (DOY 107–112; Fig. 2d), likely due to a combination of the small signal and the proximity of the red-edge transition, which makes retrieval of SIF in the O_2_-B band challenging^27^. The F_687_ to F_760_ (red to far-red) fluorescence ratio was constant at a value of ca. 0.6 during the first six days (DOY 104–109), dipped to a value of around 0.3 on the seventh day (DOY 110; when F_687_ was strongly reduced) and then rose to values between 1.2 and 1.8 (associated with the strong decrease in F_760_), before returning to pre-heat wave values at the day (DOY 113) after the end of the heat wave (data not shown). Measured SIF accounted just for 31–35% of the variability (i.e. r^2^ of linear regression) in measured midday average GPP (for perspective: air temperature explained 77% of the variability in measured midday average GPP). Simulated F_760_ and F_768_ was relatively constant throughout the heat wave (Fig. 2c,d), decreasing by 7–9% from the fourth (DOY 107) to the ninth day (DOY 112) of the heatwave.

## Discussion

Plants have developed complex regulatory mechanisms in order to optimally balance energy supply and demand by the light and dark reactions, respectively, of photosynthesis^14^. Radiative energy absorbed by chlorophyll molecules in excess of what can be used to carboxylate CO_2_, is either dissipated as heat or re-emitted as fluorescence^13^, which underlies the idea of inferring photosynthesis on the basis of measurements of chlorophyll fluorescence^28^. Even though the relationship between chlorophyll fluorescence and leaf photosynthesis is not unique^14^, tower- and satellite-based SIF measurements have been shown to scale with GPP as it changes during the season and/or with eco-climatological factors that govern the distribution of global biomes^15,17,18^. To a large degree, the explanatory power of these SIF-GPP relationships derives from the underlying significant changes in APAR across season and latitude^19^, which together with the light-use efficiency (LUE), determines GPP^29^. In the context of establishing remotely sensed SIF as a monitoring system for terrestrial GPP^30^, the robustness and sensitivity of the SIF-GPP relationship remains to be demonstrated, particularly for natural ecosystems and in situations when APAR remains constant, but the LUE changes in response to stress conditions.

In this study we have examined the SIF-GPP relationship of a Mediterranean pine forest during a naturally occurring intense heat wave in spring, when photosynthetic activity of this system is at its maximum^24^. The heat wave was short enough (9 days) so that changes in APAR (e.g. through changes in the amount/orientation of leaf area or pigment composition), inferred from near-constant NDVI measured by multiple sensors with differing field of views (Figs 1a and 2b), were negligible and alterations in SIF largely reflective of underlying changes in the partitioning of the absorbed excitation energy. The concurrent CO_2_ and COS flux measurements indicate that the decline in GPP during the early part of the heat wave was due to an increasing diffusional limitation, as stomatal closure, driven by the increasing evaporative demand, progressively decreased the uptake of both trace gases (and thus equally the enumerator and denominator of Eq. 1), resulting in a near-constant ERU (Fig. 2b). The increase in ERU during the last day of the heat wave, with surface temperatures approaching 40 °C, is interpreted to indicate a biochemical impairment of photosynthesis, causing the denominator of Eq. (1) to decrease and thus ERU to increase, similar to the increase in ERU observed at low light, when the light-independent COS uptake continues, but light-dependent photosynthesis decreases^31,32^. Limited information is available on the temperature response of the enzyme, carbonic anhydrase, ultimately responsible for the leaf uptake of COS^33^. Leaf-level gas exchange measurements, however, indicate that the COS uptake reaches its optimum at lower temperatures compared to photosynthesis^34^, which would reduce the enumerator of Eq. (1) and thus ERU, contrary to what was observed. Although ERU includes flux contributions from the soil, below canopy measurements (data not shown) indicated near zero fluxes during the measurement campaign. Even though the photosynthetic machinery appears to have suffered from severe stress conditions during the peak of the heat wave, the system is well adapted to the frequent occurrence of short-term intense heat waves as any reductions in GPP (Fig. 2a) and SIF (Fig. 2c,d) were fully and rapidly reversible^24^.

Measured F_687_, except for a 50% reduction on the seventh day of the heat wave (DOY 110), remained near constant during the entire heat wave (Fig. 2c), while F_760_ was near constant during the earlier part of the heat wave, followed by a pronounced decrease during the last two days (DOY 111–112; Fig. 2d). Near-constant SIF despite reductions in GPP can be explained by the excess energy resulting from the decline in GPP being largely dissipated via NPQ with little change in SIF^19^. During the latter part of the heat wave, when the increase in ERU suggests a biochemical impairment of photosynthesis, a clear drop in F_760_ occurred, indicating that the further decline in GPP either must have been compensated for by an even larger increase in NPQ or by alternative electron sinks which have been shown to gain importance under stress conditions (see Porcar-Castell, *et al*.^14^ and references cited therein). If that was the case, one would expect F_687_ to decrease in concert with F_760_, in contrast to what was observed (Fig. 2d). One explanation for this behaviour would be a decrease in chlorophyll content during the peak of the heat wave, which would reduce the red fluorescence emission and at the same time decrease its re-absorption and thus result in near-constant F_687_^35^. Re-absorption plays a much smaller role for F_760_, which would explain its decrease. This hypothesis conflicts with the near-constant NDVI, taken as a proxy of APAR, observed in the footprint of the SIF measurements and demonstrates the importance of additional ground measurements of leaf chlorophyll content and active fluorescence measurements (yielding key parameters such as NPQ) for interpreting canopy-scale SIF measurements^36^. The F_678_/F_760_ ratio accordingly increased markedly during the peak of the heat wave, which contrasts with Ač, *et al*.^37^, who in a meta-analysis reported evidence for a reduction of the passively measured canopy-scale red to far-red fluorescence ratio. However, the results from Ač, *et al*.^37^ are based on a single study at canopy level and the authors suggest that there are presently simply not enough studies of passively measured canopy-scale fluorescence available to defensibly discuss the red to far-red fluorescence ratio and also to what degree it may represent a sensible indicator of heat stress. We though also caution that F_687_ was much more variable even during clear sky conditions compared to F_760_, likely reflecting difficulties in retrieving SIF in the O_2_B band^27^.

SIF simulated by SCOPE overestimated F_760_ and F_687_ by 60% and 36% at the first day of the heat wave and was thus scaled to the measured value for graphical display (Fig. 2c,d). SIF values measured in this study are low compared to more productive (e.g. agricultural) ecosystems, but compare favourably with other coniferous forests^38^. We thus tend to attribute the model-data mismatch to the SCOPE model and to the uncertain parameterization of parameters such as the fluorescence quantum yield efficiency at photosystem level, which strongly controls SIF simulations, but can be quite variable across vegetation types and still is not fully characterized^39,40^. Other possible causes may be the heterogeneous 3D nature of the canopy, which SCOPE, being a 1D model^19^, is unable to account for and/or difficulties with the simulation of SIF in needleleaf canopies, even though Rossini, *et al*.^38^ successfully used SCOPE in two coniferous forests, albeit with much higher LAI. We thus scaled simulated SIF to measurements on the first day of the heat wave for graphical display (Fig. 2c,d) and constrain the discussion to the trend of simulated SIF during the course of the heat wave.

The SCOPE model simulated a weak (<10%) decline in SIF during most of the heat wave, which can be shown (see Supplement) to be the net result of a stronger simulated decline in the maximum value of the light-adapted fluorescence which over-compensated the simulated reduction in the photochemical yield (Fig. S1). The inability of the model to capture the reduction in F_760_ during the peak of the heat wave (Fig. 2c,d), suggests that simulated NPQ would need to increase more strongly as the photochemical yield declines during the heat wave or possibly that the simulated constitutive thermal dissipation does not increase enough at high temperatures^19^.

As GPP decreased strongly in an almost linear fashion during the heat wave, while F_687_ did not change much during the entire heat wave and F_760_ dropped only during the peak of the heat wave, both were poorly correlated to GPP and SIF accounted for just 31–35% of the variability in GPP. Thus, while F_760_ responded to the impairment of photochemistry during the peak of the heat wave, both F_760_ and F_687_ must be expected to exhibit limited skill in quantitatively estimating changes in GPP driven by the frequent occurrence of short-term heat waves in this region^24^ and possibly more generally during short-term climate extremes which do not go along with significant structural changes in chlorophyll content or leaf area index, and ultimately APAR. Further work is required for understanding the causes for the observed strong decline in F_760_ and the lack of in F_687_ during the peak of the heat wave, when the combined CO_2_ and COS flux measurements suggest a shift from diffusional to biochemical limitation of photosynthesis^41^. Further work is also required to transfer this empirical knowledge to models such as SCOPE, in particular with regard to the general validity of the NPQ parameterisation under stress conditions^19^ and the down-regulation of the maximum carboxylation rate at the reference temperature (V_Cmax_) during the heat wave that needed to be prescribed in the present study. This study is, to the best of our knowledge, the first to make use of joint SIF and CO_2_/COS flux measurements for diagnosing GPP and demonstrates the valuable complementary information content provided by these two independent approaches^42^.

## Methods

### Study site and period

The study site, Yatir forest, is located at the northern boundary of the Negev desert in Israel (31.35°N, 35.05°E) at an elevation of 650 m a.s.l. The forest, dominated by *Pinus halepensis* Miller, covers an area of around 2800 ha and was planted in the mid-1960s. Stand density is ca. 300 trees ha^−1^, the leaf area index amounts to ca. 1.5 m^2^ m^−2 ^^43^. The soil has been described as a light brown 0.25–1 m deep Rendzina^26^. The climate is Mediterranean with an average annual temperature and rainfall of 18.2 °C and 280 mm, respectively.

While the site is active since 2000^26^, measurements reported here were conducted during a campaign from the beginning of March to the end of May 2017, which covers the transition from peak photosynthetic activity in spring to the beginning of the extended summer drought. During this campaign, measurements of sun-induced fluorescence were conducted during a limited period in April, within which an eight-day heat wave (DOY 104–112) was observed.

### Ecosystem-scale flux and ancillary measurements

Ecosystem-scale fluxes of COS, CO_2_, H_2_O and energy were measured by means of the eddy covariance method^44^. The three wind components and the speed of sound were measured using a three-dimensional sonic anemometer (R-50, Gill, UK). COS, CO_2_ and H_2_O mixing ratios were quantified using a quantum cascade continuous wave laser (QCL) absorption spectrometer (QC-TILDAS-CS, Aerodyne, USA) at a wavenumber of ca. 2056 cm^−1^. CO_2_ and H_2_O flux measurements were validated against the long-term continuous flux measurements at the site using an infrared gas analyser (Li-7000, LiCor, USA; see^22^). The QCL and associated hardware (thermo cube and vacuum pump) were housed in climate-controlled instrument huts at the base of the tower. Sample air was drawn from the inlet (close to the sonic anemometer) through 25 m heated (ca. 5° above ambient) PFA Teflon tubing (4 mm inner diameter) through a filter (1–2 µm, PTFE) to the QCL at a flow rate of ca. 6.5 l min^−1^. During the last 2 minutes of every half-hour, zero-air was switched into the QCL in order to determine stability of the instrument zero. Calibration gas, traceable to NOAA, was used periodically to check the instrument span. The QCL was operated at a pressure of ca. 3.3 kPa using a built-in pressure controller and temperature of the optical bench and housing controlled to 30 °C. Fitting of absorption spectra at 5 Hz, storing of calculated COS, CO_2_ and H_2_O dry mole fractions, switching of zero/calibration valves, control of pressure lock and other system controls were realised by the TDLWintel software (Aerodyne, USA) run on a PC synchronised with the PC collecting anemometer data using the NTP software (Meinberg, Germany). The two data streams were then merged and aligned in time during post-processing using proprietary software^32^.

Using the post-processing software EdiRe (University of Edinburgh), eddy fluxes of COS, CO_2_, H_2_O and energy were calculated as the covariance between turbulent fluctuations of the vertical wind speed and the scalar mixing ratios derived from Reynolds averaging of 28 min blocks of data. The co-ordinate system’s vector basis was aligned with the mean wind streamlines using the 2D rotation^45^. The, mostly tube-induced, time delay between the wind components and the COS, CO_2_ and H_2_O mixing ratios was determined by identifying the maximum/minimum of the cross-correlation function. Frequency response corrections were applied to raw eddy fluxes accounting for low-pass and high-pass filtering^32^. The net ecosystem exchange of COS, CO_2_, H_2_O and energy was then calculated as the sum of the corrected vertical eddy covariance term and the storage flux, the latter being estimated from the rate of change in scalar concentration at the reference height.

Half-hourly flux data were subject to a series of quality control tests^46^ and filtered for the potential underestimation during nighttime periods of low turbulence^47^. Gap-filling and flux partitioning of NEE calculated based on the infrared gas analyser measurements, was conducted as described in detail in Tatarinov, *et al*.^24^.

The major environmental parameters required for the interpretation of COS and CO_2_ fluxes and as input for the modelling (see below) were measured continuously at the site and included the following parameters: Down- and up-welling radiation above the canopy and the soil surface (photosynthetically active, shortwave and longwave radiation), air temperature and humidity, soil temperature, water content and heat flux, static air pressure.

### Proximal and remote sensing

Sun-induced fluorescence in the red (O_2_-B band, 687 nm) and far-red (O_2_-A band, 760 nm), referred to as F_687_ and F_760_ respectively, regions was measured in a hemispherical-conical configuration using the fluorescence box (FLOX) instrument (JB Hyperspectral Devices, Düsseldorf, Germany), a field-proven^48^ home-build system consisting of a high-resolution (0.31 nm FWHM) thermo-electrically cooled spectrometer (QE Pro, Ocean Optics, USA; 648–808 nm) housed in a thermally regulated field enclosure. An upward facing fibre cable equipped with a cosine diffuser measured the down-welling irradiance, the up-welling radiance was measured with a bare fibre (25° FOV) pointing North at a pine tree crown some 5 m distance from the tower at a zenith angle of ca. 30°. Both fibres were connected through inline shutters with a bi-furcated fibre to the spectrometer. A spectral and radiometric calibration of the complete system was conducted after the field campaign using appropriate light sources traceable to international standards (NIST). Apparent reflectance was calculated from alternating measurements of down- and up-welling (ir)radiance with the integration time optimised for the signal-to-noise ratio of the instrument, each followed by a corresponding dark current measurement. SIF was calculated from these data using the so-called spectral fitting method^49^.

The FLOX instrument also includes a VIS-NIR spectrometer, whose data however could not be used due to a broken cosine diffuser on the upward looking optical fibre cable. Instead we have calculated the normalized difference vegetation index (NDVI) based on the data of the hyperspectral spectrometer that was also used to measure SIF and thus covers the same field of view. NDVI was computed using the mean reflectance factors for the spectral range centred at 800 ± 5 nm for the reflectance in the near infrared and at 670 ± 5 nm. The NDVI was additionally measured from the same tower using narrow-band sensors sensitive in the red (659 nm) and near-infrared region (858 nm) (SKR 1850, Skye Instruments, UK). Narrow-band sensors pointed South and, in contrast to the FLOX system, viewed a mix of tree crowns and background (bare soil), as the downward looking sensors were equipped with a cosine diffuser. In order to track vegetation greenness in the larger footprint of the flux tower, MODIS collection 6 NDVI was downloaded from ORNL DAAC (https://daacmodis.ornl.gov/cgi-bin/MODIS/global/subset.pl) for a 250 × 250 m area around the flux tower.

### Simulation modelling

The soil-vegetation-atmosphere exchange of CO_2_ and COS, as well as top-of-canopy sun-induced fluorescence were simulated with the Soil-Canopy-Observation of Photosynthesis and Energy fluxes (SCOPE) model (version 1.7 downloaded from https://github.com/Christiaanvandertol/SCOPE)^19,25,50^, which has been instrumental in SIF-related research during recent years^40,51–53^ and has been successfully used for a pine forest previously^38^. SCOPE is a 1D multi-layer model which computes leaf processes (photosynthesis, stomatal conductance, transpiration, reflection, transmission, emission of radiation) and integrates these processes at the canopy-scale to yield above-canopy fluxes of CO_2_, H_2_O and energy, as well as reflected and emitted radiation. Simple calculations of the soil energy balance provide a lower boundary condition for the canopy model. The upper boundary is represented by measured meteorological inputs (incident short- and long-wave radiation, static air pressure, air temperature and relative humidity, wind speed and CO_2_ mole fraction) at the reference height some distance above the canopy.

While a full description of the model is beyond the scope of this study, we provide a narrative description of the key modules involved in the simulation of canopy photosynthesis and SIF below and refer to the original model descriptions for further information.

Three major model components interact in the simulation of canopy photosynthesis and SIF: (i) The biochemical model simulates leaf gross and net photosynthesis by combining a mechanistic model of C_3_ photosynthesis^54^ with an empirical model of stomatal conductance^55^. The energy demand by photosynthesis, i.e. photochemical quenching, is then combined with an empirical model of NPQ to yield the fraction of absorbed light re-emitted as fluorescence^19^. Here we have used the option of the drought-stress parameterisation of K_N_, the rate coefficient for non-photochemical quenching (Fluorescence_model = 0). (ii) The leaf-scale radiative transfer model FLUSPECT^50^ simulates leaf absorption, transmission and reflection of radiation (400–2500 nm with 1 nm resolution) and calculates the probability, separately for the upper and lower side of the leaf, of absorbed photosynthetically active radiation being returned as fluorescence (640–850 nm). Here we have used a new option in version 1.7 of SCOPE to simulate the fluorescence spectrum as a whole (calc_PSI = 1), not separating photosystem I and II contributions as earlier versions did. (iii) Within-canopy transport of incident radiation and leaf-emitted fluorescence is then achieved by the Scattering of Arbitrarily Inclined Leaves (SAIL) model^56^.

In order to compare F_760_ and F_687_ with the model simulations, simulated directional SIF was averaged over the 759–761 nm and 686–688 nm range, respectively. SIF simulated by SCOPE overestimated F_760_ and F_687_ by 60% and 36% at the first day of the heat wave and was thus scaled to the measured value for graphical display (Fig. 2c,d). Soil respiration, not included in the standard version of SCOPE, was simulated using a site-specific parameterisation^57^. Standard model parameters were used except for the following, which were either measured at the study site previously or were determined specifically for *Pinus halepensis* under Mediterranean climatic conditions as detailed in Table 1. SCOPE is a static model, i.e. changes in canopy structure and function in response to phenological development or extreme events, need to be prescribed. In order to mimic a progressive limitation of photosynthesis during the heat wave^58^, the maximum carboxylation rate at the reference temperature (V_Cmax_), was linearly decreased from 45 to 30 µmol m^−2^ s^−1^ over the duration of the heat wave, consistent with measurements of light-saturated net photosynthesis of *Pinus halepensis* at the study site^59^ and then set back to 45 µmol m^−2^ s^−1^ at the day after the end of the heat wave.

**Table 1:** SCOPE model parameters.

Abbreviation	Parameter	Units	Value	Reference
C_ab_	Chlorophyll a & b content	µg cm^−2^	35	^60^
C_ca_	Carotenoid content	µg cm^−2^	25% of C_ab_	
C_dm_	Dry matter content	g cm^−2^	0.023	
C_w_	Equivalent leaf water layer	cm	0.0023	^61^
C_s_	Senescent material fraction	fraction	0	
C_ant_	Anthocyan content	µg cm^−2^	0	
N	Leaf thickness parameter	—	1.5	
fqe	Fluorescence emission efficiency	—	0.01	
V_Cmax_	Maximum carboxylation rate at reference temperature	µmol m^−2^ s^−1^	30–45	^62^
m	Stomatal conductance parameter	—	6	^62^
R_dparam_	Dark respiration as fraction of V_Cmax_	fraction	0.0055	^63^
LAI	Leaf area index	m^2^ m^−2^	1.5	^62^
z, h_c_	Measurement and canopy height	m	18, 11	^62^
LIDF_a_, LIDF_b_	Leaf inclination distribution	—	−0.35, −0.15	
w	Leaf width	m	0.001	

### Code Availability

The analyses were conducted in Matlab (R2016b, Mathworks, USA) and the corresponding scripts are available from the corresponding author upon reasonable request.

## Electronic supplementary material

Supplementary Information

## Data Availability

The data used in this study are available from the corresponding author upon reasonable request.
