# Supplementary material for: Sun-induced fluorescence and gross primary productivity during a heat wave
Source: Sci Rep. 2018 Sep 21;8:14169. doi: 10.1038/s41598-018-32602-z (PMC6155073; doi:10.1038/s41598-018-32602-z)
Supplement: Supplementary file 1 — Supplementary Information [file 41598_2018_32602_MOESM1_ESM.docx]

Sun-induced fluorescence and gross primary productivity during a heat wave

**Supplementary material**

*Authors:*

G. Wohlfahrt^1*^, K. Gerdel^1^, M. Migliavacca^2^, E. Rotenberg^3^, F. Tatarinov^3^, J. Müller^3^, A. Hammerle^1^, T. Julitta^4^, F. M. Spielmann^1^ and D. Yakir^3^

Affiliations:

^1^ University of Innsbruck, 6020 Innsbruck, Austria.

^2^ Max Planck Institute for Biogeochemistry, 07745 Jena, Germany.

^3^ Weizmann Institute of Science, 76100 Rehovot, Israel.

^4^ University of Milano-Bicocca, 20126 Milan, Italy.

^*^ e-mail: [georg.wohlfahrt@uibk.ac.at](mailto:georg.wohlfahrt@uibk.ac.at)

**Temperature response of leaf-level fluorescence in SCOPE**

In SCOPE^1^, the fluorescence yield of a light-adapted leaf under steady-state conditions (*Ф_Ft_*) is simulated by re-arranging the equation put forward by Genty, et al. ^2^, i.e.

$\Phi_{Ft}=\Phi_{F'm}\left( 1-\Phi_{P} \right)$, (1)

where *Ф_P_* represents the photochemical yield and *Ф_F’m_* the fluorescence yield of a light-adapted leaf under steady-state conditions after a saturating light pulse has been applied^1^.


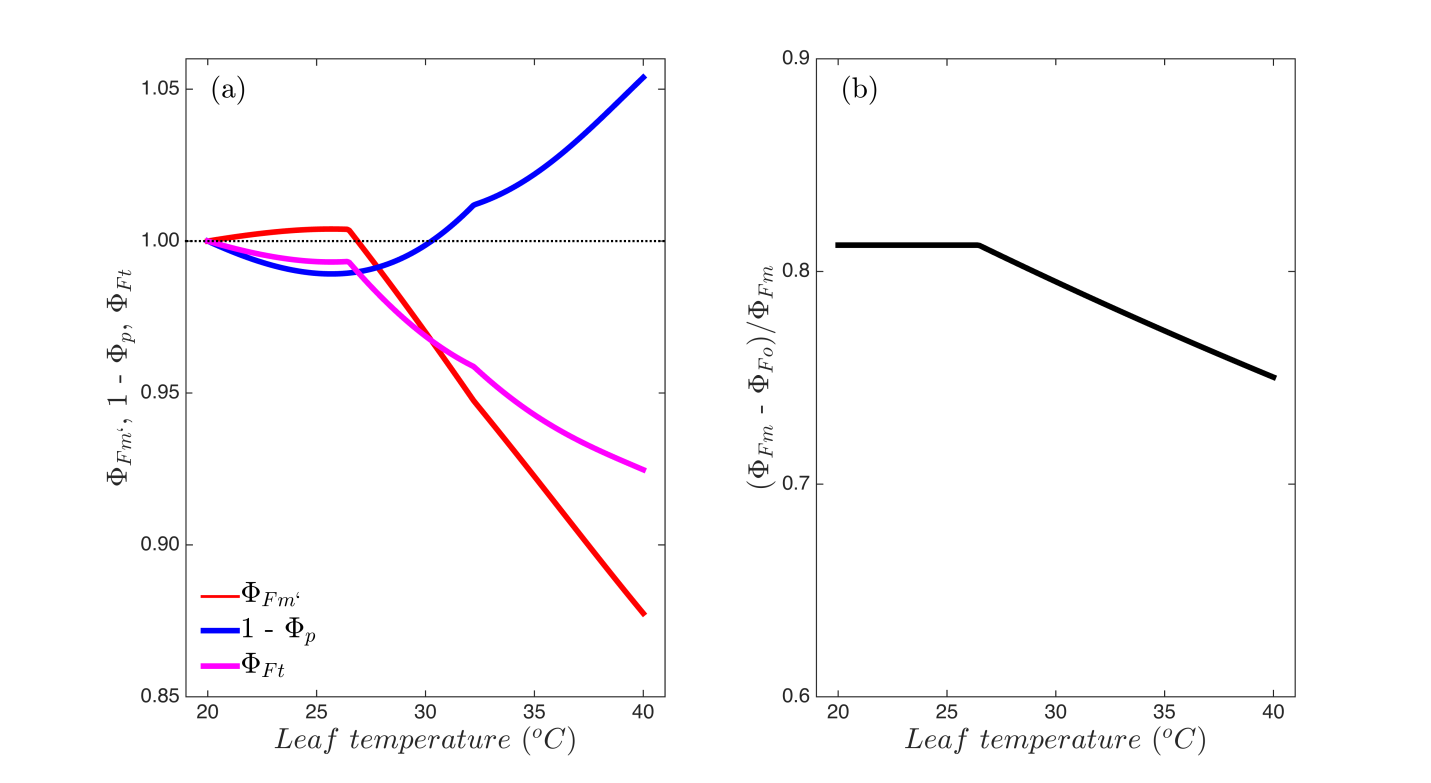


Figure S1. Simulated temperature response of (a) the steady-state fluorescence yield and its component processes (all normalized to their values at 20°C) and (b) the variable to maximum dark-adapted fluorescence yield. Simulations were conducted with the calibrated leaf-scale module of SCOPE by varying leaf temperature between 20°C and 40°C, corresponding to the temperatures at the start and peak of the heat wave respectively, and an accompanying linear decrease of the maximum carboxylation rate from 45 to 30 µmol m^‑2^ s^‑1^. Absorbed photosynthetically active radiation was set to 2000 µmol m^‑2^ s^‑1^.

The latter is a function of the rate coefficients for fluorescence (*K_F_*) and constitutive (*K_D_*) and energy-dependent (*K_N_*) thermal dissipation, i.e.

$\Phi_{F'm}={K_{F}}/\left( K_{F}+K_{D}+K_{N} \right)$. (2)

In SCOPE *K_F_* is taken as a constant, *K_D_* is temperature-dependent and *K_N_* is a function of the relative degree of light saturation of photosynthesis and thus *Ф_P_*^1^. Information on *Ф_P_* is supplied by a combined model of leaf photosynthesis and stomatal conductance^3^, i.e.

$\Phi_{p}={J_{e}}/{J_{aPAR}}$, (3)

where *J_e_* stands for the electrons effectively used to carboxylate CO_2_ and *J_aPAR_* for the PAR absorbed by photosystem II.

The temperature response of steady-state fluorescence thus depends on the temperature response of the two components, *Ф_F’m_* and (1 - *Ф_P_*), which are shown in Figure S1a. The term (1 ‑ *Ф_P_*) exhibited an inverted optimum shape with a minimum around 26°C, reflecting the competing influence of the underlying processes which decrease (the RUBISCO specificity factor and the photochemical yield of a dark-adapted leaf) or increase (the Michaelis-Menten constants of carboxylation and oxygenation) with temperature or show an optimum-type response (the maximum rate of carboxylation). *Ф_F’m_* increased with temperature until 26°C, driven by the increase of *Ф_P_* with temperature, which in turn decreased K_N_ (Eq. 3). At higher temperatures, the increase of *K_D_* with temperature caused *Ф_F’m_* to decline. The net result of these two opposing shapes is (i) that the response of SIF to temperature was dampened^1^, and (ii) that the reduction in *Ф_P_* with increasing temperature was over-compensated by a temperature-mediated reduction in *Ф_F’m_*, resulting in an overall decrease of *Ф_Ft_* by around 7 % between 20° and 40°C (Fig. S1a). For comparison with the huge body of literature in which active measurements of chlorophyll fluorescence were used for diagnosing photosynthetic stress, we include the simulated temperature response of the variable to maximum fluorescence of dark adapted leaves^4^ in Figure S1b. This widely used parameter was constant at a value (0.82) thought to reflect unstressed conditions^4^ until around 26°C and then decreased by 8 % until 40°C leaf temperature.

**References**

1 van der Tol, C., Berry, J. A., Campbell, P. K. E. & Rascher, U. Models of fluorescence and photosynthesis for interpreting measurements of solar-induced chlorophyll fluorescence. *Journal of Geophysical Research: Biogeosciences* **119**, 2014JG002713, doi:10.1002/2014JG002713 (2014).

2 Genty, B., Briantais, J.-M. & Baker, N. R. The relationship between the quantum yield of photosynthetic electron transport and quenching of chlorophyll fluorescence. *Biochimica et Biophysica Acta* **990**, 87-92, doi:10.1016/s0304-4165(89)80016-9 (1989).

3 Collatz, G. J., Ball, J. T., Grivet, C. & Berry, J. A. Physiological and environmental regulation of stomatal conductance, photosynthesis and transpiration: a model that includes a laminar boundary layer. *Agric. For. Meteorol.* **54**, 107-136, doi:10.1016/0168-1923(91)90002-8 (1991).

4 Maxwell, K. & Johnson, G. N. Chlorophyll fluorescence--a practical guide. *J. Exp. Bot.* **51**, 659-668, doi:10.1093/jexbot/51.345.659 (2000).
